# Differential Effects of One Meal per Day in the Evening on Metabolic Health and Physical Performance in Lean Individuals

**DOI:** 10.3389/fphys.2021.771944

**Published:** 2022-01-11

**Authors:** Emma C. E. Meessen, Håvard Andresen, Thomas van Barneveld, Anne van Riel, Egil I. Johansen, Anders J. Kolnes, E. Marleen Kemper, Steven W. M. Olde Damink, Frank G. Schaap, Johannes A. Romijn, Jørgen Jensen, Maarten R. Soeters

**Affiliations:** ^1^Department of Endocrinology and Metabolism, Amsterdam Gastroenterology Endocrinology Metabolism, Amsterdam University Medical Centers—Location AMC, University of Amsterdam, Amsterdam, Netherlands; ^2^Department of Physical Performance, Norwegian School of Sport Sciences, Oslo, Norway; ^3^Section of Specialized Endocrinology, Department of Endocrinology, Oslo University Hospital, Oslo, Norway; ^4^Hospital Pharmacy, Amsterdam University Medical Centers—Location AMC, University of Amsterdam, Amsterdam, Netherlands; ^5^Department of Surgery, NUTRIM School of Nutrition and Translational Research in Metabolism, Maastricht University, Maastricht, Netherlands; ^6^Department of General, Visceral and Transplantation Surgery, RWTH University Hospital Aachen, Aachen, Germany; ^7^Department of Internal Medicine, Amsterdam University Medical Centers—Location AMC, University of Amsterdam, Amsterdam, Netherlands

**Keywords:** time restricted eating, time restricted feeding, physical performance, postprandial metabolism, bile acids, human, glucose, energy expentidure

## Abstract

**Background:** Generally, food intake occurs in a three-meal per 24 h fashion with in-between meal snacking. As such, most humans spend more than ∼ 12–16 h per day in the postprandial state. It may be reasoned from an evolutionary point of view, that the human body is physiologically habituated to less frequent meals. Metabolic flexibility (i.e., reciprocal changes in carbohydrate and fatty acid oxidation) is a characteristic of metabolic health and is reduced by semi-continuous feeding. The effects of time-restricted feeding (TRF) on metabolic parameters and physical performance in humans are equivocal.

**Methods:** To investigate the effect of TRF on metabolism and physical performance in free-living healthy lean individuals, we compared the effects of eucaloric feeding provided by a single meal (22/2) vs. three meals per day in a randomized crossover study. We included 13 participants of which 11 (5 males/6 females) completed the study: age 31.0 ± 1.7 years, BMI 24.0 ± 0.6 kg/m^2^ and fat mass (%) 24.0 ± 0.6 (mean ± SEM). Participants consumed all the calories needed for a stable weight in either three meals (breakfast, lunch and dinner) or one meal per day between 17:00 and 19:00 for 11 days per study period.

**Results:** Eucaloric meal reduction to a single meal per day lowered total body mass (3 meals/day –0.5 ± 0.3 vs. 1 meal/day –1.4 ± 0.3 kg, *p* = 0.03), fat mass (3 meals/day –0.1 ± 0.2 vs. 1 meal/day –0.7 ± 0.2, *p* = 0.049) and increased exercise fatty acid oxidation (*p* < 0.001) without impairment of aerobic capacity or strength (*p* > 0.05). Furthermore, we found lower plasma glucose concentrations during the second half of the day during the one meal per day intervention (*p* < 0.05).

**Conclusion:** A single meal per day in the evening lowers body weight and adapts metabolic flexibility during exercise via increased fat oxidation whereas physical performance was not affected.

## Introduction

Most humans spend more than 12–16 h per day in the postprandial state. Generally, food intake occurs in a three meal per 24 h fashion with in-between meal snacking (Kant, 2018; Paoli et al., 2019). After meal ingestion, plasma insulin concentrations rise and subsequently increase glucose uptake and -oxidation in peripheral tissues (Thiebaud et al., 1982). Furthermore, insulin activates lipoprotein lipase which promotes the storage of dietary fat in adipose tissue (Lafontan and Langin, 2009). Lowering of plasma insulin concentrations during fasting (e.g., overnight) permits lipolysis, a shift from glucose to fat oxidation resulting in a state of physiological insulin resistance if the fasting is continued (Lafontan and Langin, 2009; Soeters et al., 2012).

Several postprandial signals (e.g., plasma bile acids, fibroblast growth factor 19 (FGF19), lipids) are still increased in the systemic circulation 4–5 h after meal ingestion (Schrezenmeir et al., 1993; Van Nierop et al., 2019; Meessen et al., 2020). From an evolutionary point of view it may be reasoned that the human body is habituated to a lower meal frequency (Meiselman, 2000). Metabolic flexibility (i.e., reciprocal changes in carbohydrate- and fatty acid oxidation) is a characteristic of metabolic health. Continuous/frequent food intake on the other hand may result in reduced metabolic flexibility, obesity and type 2 diabetes mellitus (T2DM) (Smith et al., 2018). These lifestyle-related diseases are associated with modern eating habits and a sedentary lifestyle (Kopp, 2019). Nutritional- and exercise interventions should be the first in prevention- or treatment strategies because of the biological rationale, low cost and non-pharmacological approach (Manson et al., 2004).

The most used strategies of intermittent fasting can be clustered into three groups: alternate day fasting, whole- day fasting and time-restricted feeding (TRF). Alternate day fasting incorporates alternating *ad libitum* feeding days and fasting days (accompanied with 1 small meal), whilst the whole-day fasting strategy involves 1 or 2 days of complete fasting per week plus and *ad libitum* feeding on the residual days (Tinsley and La Bounty, 2015). TRF encompasses many different strategies, but is mainly the combination of a limited feeding time-window and “prolonged” fasting (Patterson and Sears, 2017; McAllister et al., 2020). As a result of the wide range of TRF protocols in studies (due to variation in meal timing, duration of fasting time, with or without caloric restriction), the effects of TRF on metabolic parameters and physical performance are scarce and equivocal (Moon et al., 2020; Pellegrini et al., 2020). Some metabolic studies show that TRF may improve whole-body insulin sensitivity whereas other studies reported no effect on glucose metabolism or even reported impaired glucose tolerance (Halberg et al., 2005; Carlson et al., 2007; Soeters et al., 2009; Cienfuegos et al., 2020; Jones et al., 2020). Interestingly TRF (16/8) decreased fat mass without affecting strength in resistance-trained males (Moro et al., 2016).

Therefore, we investigated the physiological effects of eucaloric TRF (22/2, one meal per day in the evening) for 11 days on metabolic health and physical performance in free-living healthy lean individuals compared to normal food intake (three meals per day) in a crossover design.

## Materials and Methods

### Participants

We initially included 13 healthy individuals and inclusion criteria were a body mass index (BMI) between 20 and 30 kg/m^2^, and/or fat mass between 12 and 30% and some training experience. Exclusion criteria were a history of cardiovascular disease, diabetes mellitus (type 1 or type 2), eating disorders, mental disorders, other diseases which may affect metabolism, cholecystectomy, smoking and already on an intermittent fasting pattern (self-reported). Participants were not allowed to consume alcohol or smoke tobacco during the study. We did not control this study for sex. The study was approved by the Ethics Committee at Norwegian School of Sport Sciences (Application #56-190618). Oral and written informed consent were obtained according to the Declaration of Helsinki (2013). The study was not prospectively registered in a trial registry.

### Study Design and Intervention

The study was conducted from September 2018 until December 2018 at the Norwegian School of Sports Sciences in Oslo, Norway. The study had a randomized crossover design with two study periods of 11 days of TRF. Participants consumed all the calories needed for a stable weight in either three meals per day (3 meals/day, breakfast, lunch and dinner) or one meal per day (1 meal/day, between 17:00 and 19:00). A 2-week wash-out period was included between the study periods. During both study periods, participants were allowed to consume unlimited water, coffee or tea (without sugar/honey). Only artificial sweeteners were allowed. To maintain a stable weight during the intervention periods, the daily energy need was determined based on gender, age, weight, height and activity pattern with the Norwegian Health Information AS (Beregning av kaloribehov—NHI.no, 2021). Participants individually designed to diet to reach the energy requirement. Energy requirement was calculated from body composition determined with DXA according to Mifflins formula (Mifflin) with correction for activity factor (PAL) (Shetty et al., 1988). Participants used the App from Lifesum (Lifesum AB, v6.3.10, Stockholm, Sweden) for registration and calculation of energy in the daily food intake. Dietary data were reported to test leaders for day 5, 8, and 11. Macronutrient intake was not controlled. Participants practiced all physical performance tests. The investigators were aware of the dietary intervention. We have tested reliability for VO_2m*ax*_ in elite athletes with a coefficient of variance (CV) of 1.8%. We have tested reliability for jumping height for trained males and students with experience with jump training from sport, and again have CV as low as 2–3%. We have not tested reliability for maximal fat oxidation. We have not tested CV for maximal isokinetic strength but the variation has been reported to 8% (Duarte et al., 2018).

### Assessment of Metabolic Health

#### Body Composition

Body composition was measured before (day 1) and at day 11 of each study period in fasted condition (no liquids or solid food) at the morning (07:00–11:00) with dual-energy X-ray absorptiometry (DXA) (Prodigy Advance PA + 302147, Lunar, San Francisco, CA, United States) including total body mass, fat mass, fat free mass (FFM) and lean body mass [FFM *minus* bone mineral content (BMC)]. We have measured body composition (DXA.) twice before a 7 days fasting study. The CV for FFM (kg) was 0.98% (*n* = 13) and for body fat (kg) was 1.16%.

#### Continuous Glucose Monitoring

Participants carried a Dexcom G4^®^ PLATINUM Continuous Glucose Monitoring (CGM) System (Dexcom, Inc., San Diego, United States) for continuous measurement of subcutaneous glucose concentrations from days 5 to 11. We calculated the mean glucose level per hour and used these values for the statistical analysis.

#### Resting Energy Expenditure

At day 11, after an overnight fast, participants arrived at the research facility in the morning (between 07:00 and 10:00). Resting energy expenditure (REE) was measured by indirect calorimetry (Oxycon Pro, Jager Instr; Hoechberg, Germany) with breath by breath software (LABManager 5.3.0.4) in a supine position for 15 min using a mask (V2 Mask, Hans Rudolph Inc., Shawnee, Kansas, United States). Oxygen volume (VO_2_) (mL/min), carbon dioxide volume (VCO_2_) (mL/min) were measured. Respiratory quotient (RQ) and REE (kcal/day) were calculated as follows: RQ = (VCO_2_)/(VO_2_) and REE = (3.941 * VO_2_ (L/min) + (1.11 * VCO_2_ (L/min) × 1,440 min (modified Weir Equation) (Weir and de, 1949).

#### Mixed Meal Test

At day 12, mixed meal tests (MMT) were performed in the morning to assess the effects of meal reduction on postprandial plasma glucose, insulin, bile acid and FGF19 responses. After an overnight fast, participants entered the research facility and a cannula was placed into an antecubital vein for blood withdrawal. We used Nutridrink Compact (Nutricia, Zoetermeer, The Netherlands, macronutrient composition: 49% carbohydrates, 35% fat and 16% protein) as liquid mixed meal. Participants consumed 25% of their measured REE. Venous blood was sampled at time point (T) 0 (fasting) and at T30, T60, T90, T120, T150, T180, and T240 min after meal ingestion. Blood samples were collected in EDTA or serum tubes (Greiner Bio-One, Kremsmunster, Austria). Serum tubes were kept at room temperature for 30 min. The EDTA tubes were directly stored on ice, centrifuged (10 min; 4^°^C; 2,500 g) and stored at –80° until laboratory analysis. Homeostatic Model Assessment for Insulin Resistance (HOMA-IR) was used to quantify insulin resistance [calculated as (fasting plasma glucose * fasting plasma insulin)/135)]. We calculated the Matsuda index to express changes in whole-body insulin sensitivity (Matsuda and DeFronzo, 1999). Plasma glucose concentrations assessed during the MMT were measured with a blood glucose meter (HemoCue^®^ Glucose 201 + System). Serum insulin concentrations were analyzed with Immulite 2000 (Siemens, Healthcare Diagnostics, Breda, The Netherlands). Plasma total bile acid and FGF19 levels were measured with an enzymatic method (Diazyme Laboratories, Inc., Poway, California, United States) and in-house developed ELISA, respectively (Schaap et al., 2009). Plasma cholesterol, low-density lipoprotein cholesterol (LDL-C), high-density lipoprotein cholesterol (HDL-C), triglycerides (TGs), alanine amino transferase (ALT) and aspartate amino transferase (ALT) were measured at Fürst Laboratory, Oslo, Norway (Advia Centaur XPT, Siemens Medical Solutions Diagnostics, Tokyo, Japan with analytical coefficient of variation of 2.3% for ALT and 3.3% for AST).

### Assessment of Physical Performance

Participants did not exercise the day before the physical performance tests and tests were performed after an overnight fast.

#### Physical Activity

Participants carried an accelerometer (ActiGraph GT1M; Actigraph, Pensacola, United States) at the hip during both intervention periods from days 5 to 11. Participants were only allowed to remove the accelerometer during water activities or sleep. Data from the Actigraph were analyzed with ActiLife version 6.13.3 (ActiLife software, ActiGraph, LLC, Pensacola, Florida, United States). We included data for analyses when more than 400 min for at least 3 days were registered (in total for 8 participants). The data analyses procedure obtained from the ActiGraph has been published in detail elsewhere (Hansen et al., 2012). In short, the accelerometer detects vertical acceleration in units named counts and collects data at a rate of 30 times/s. Counts per minute (CPM) is a quantity of total physical activity and was calculated as the total number of counts for the valid period divided by the total wearing time. CPM thresholds of different intensities (matches the energy costs of the given intensity) were applied to the dataset (sedentary activity < 100 CPM, low-intensity 100-2019 CPM, and moderate-to-vigorous physical activity (MVPA) is ≥ 2020 CPM) (Hansen et al., 2012).

#### Cycle Tests: Fat Oxidation, Wingate and Maximal Oxygen Uptake Tests

At day 10, the cycle tests were performed on an ergometer bike (Excalibur Sport with Pedal Force Measurement, Lode, Groningen, The Netherlands). Heart rate was measured with the Polar RS800CX watch (Polar Electro Oy, Kempele, Finland). Again VO_2_ (mL/min) and VCO_2_ (mL/min) were measured with indirect calorimetry, but during the cycle tests a mouthpiece and nose clip were used (2,700 series, Hans Rudolph Inc., Shawnee, Kansas, United States).

Before the start of the study periods, participants conducted an incremental test to assess the relationship between load (watt) and oxygen uptake (VO_2_) in order to calculate the loads for the fat oxidation and maximal oxygen uptake (VO_2m*ax*_) test (Dahl et al., 2020).

**FIGURE 1:**
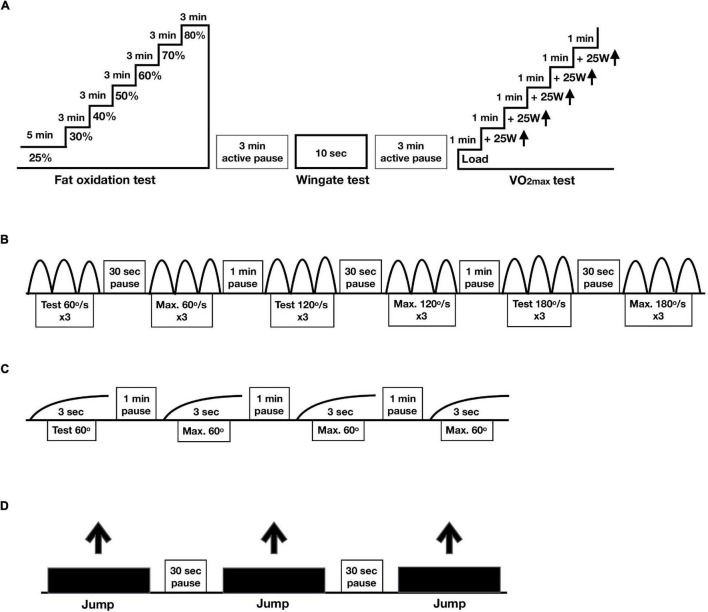
Protocols physical performance. In a randomized crossover design, 11 healthy participants consumed all the calories needed for a stable weight in either three meals per day (3 meals/day, breakfast, lunch and dinner) or one meal per day (1 meal/day, between 17:00 and 19:00) for 11 days. **(A)** Protocol cycle tests including the fat oxidation (% of VO_2m*ax)*_, Wingate and VO_2m*ax*_ tests. **(B)** Protocol for maximal isokinetic strength, **(C)** protocol for maximal isometric strength and **(D)** protocol for maximal jumping height.

First, the fat oxidation test was conducted (Figure 1A). Oxygen uptake was measured during the entire test (21 min) and metabolic data from the last min of the 6 loads (30, 40, 50, 60, 70, and 80% of VO_2m*ax*_) was used in the calculations of maximal fat oxidation (Andersson Hall et al., 2016). Fat oxidation (gram/min) was assessed with the formula 1.67 *VO_2_ (L/min) –1.67*VCO_2_ (L/min) and carbohydrate oxidation (gram/min) with VCO_2_*4.55 – VO_2_*3.21 (L/min) (Frayn, 1983). The Frayn equation assumes that protein oxidation is neglible in healthy persons. Since our study involved only healthy participants, we did not include protein oxidation in our analyses. During each stage, plasma lactate was determined. Lactate concentrations were obtained via bedside measurements (Safe-T-Pro Plus, Accu-Check, Roche, Basel, Switzerland) drawing the blood into a 20 μl sodium heparin glass tube (Plastic capillsaries end-to-end, EKF Diagnostic, Barleben, Germany) and then analyzed (Biosen C-Line, EKF Diagnostic, Barleben, Germany). Before testing, the Biosen C-Line was calibrated using a standardized lactate. Perceived exhaustion was scored with the BORG scale (Borg, 1982).

Second, to assess maximal anaerobic power production (watt) in 10 s, the modified Wingate test was performed (Sollie et al., 2018; Correia et al., 2021). The Wingate test was perform on a Lode ergometer bike (Lode Exacalibur Sport, Gronnigen, NL) with a torque factor of 0.8. Maximal power during the 10 s test is reported. Prior to the 10 s of cycling at maximal power, there was a 30 s countdown during which participants were instructed to increase the revolutions per minute (RPM) to approximately 115. Then, participants were asked to give maximum output for 10 s. The resistance of the bike ergometer was automatically increased based on the power of the participants. The participants were encouraged to perform maximal at the Wingate tests. The 10-s modified Wingate test in relatively easy to complete for the participants as no major exhaustion develops.

After 3 min of rest, the VO_2m*ax*_ test was performed (Rustad et al., 2016; Sollie et al., 2018; Correia et al., 2021). The load was increased with 25 watt every minute until voluntary exhaustion or RPM < 60. Capillary blood samples were drawn for the measurement of lactate and rate of perceived exertion was rated on the BORG scale (Borg, 1982).

#### Strength

The assessment of strength was performed at days 1 and 10. Maximal isokinetic strength (power) in knee extensors (quadriceps femoris) and knee flexors (hamstrings) was tested in the dominant leg at three different velocities in dynamometer (60, 120, and 180°/s) (Equipment - Humac dynamometer, NORM, Computer Sports Medicine Incorporated (CSMi), Stoughton, MA, United States) (Figure 1B; Habets et al., 2018). Participants performed two sets of three repetitions on every velocity, from which only the best set of repetitions was used for analysis.

Maximal isometric strength was tested at 60° angle during 3 s. After trying out the movement, participants performed three attempts from which we used the best one for analysis (Figure 1C).

Jumping height (in centimeters) was measured by performing a countermovement jump on a force platform (Hur Labs Oy, Tampere, Finland) with usage of Force Platform Software Suite (Figure 1D). After a 10-min warming-up on an exercise bike, participants started from a standing position, initiated a downward movement, which was immediately followed by an upward movement leading to the jump. The participants were asked to position their hands on the hips in a position comfortable for jumping. Three jumps were performed, from which we used the best attempt for analysis (Tangen et al., 2020).

### Power Calculation

This study was designed as an observational study to investigate the effects of TRF on metabolic health and physical performance. The statistical power calculation was based on the plasma fasting glucose results from a crossover study after a 40 h fast in healthy participants (Van Nierop et al., 2019). It pointed out that at least 10 participants needed to finish the study, to have 80% power (α = 0.05, paired, two-sided test) to detect a 0.5 mmol/L difference in fasting glucose concentrations (standard deviation of differences 0.63 mmol/L).

### Statistical Analyses

The distribution of data was analyzed with the Shapiro-Wilk test. Comparisons between the intervention periods (1 meal/day vs. 3 meals/day) were made with a paired *T*-test when data was normally distributed or otherwise the Wilcoxon sign-ranked test was used. Differences between study periods (1 meal/day vs. 3 meals/day) in combination with pre- (day 1) and post- (day 2) measurements within the intervention period were analyzed with the two-way repeated measurements ANOVA (Strength). We also used the two-way repeated measures ANOVA for the analyses of the CGM data and the MMT data (glucose insulin, total bile acids and FGF19). The Bonferroni correction was applied for multiple comparisons. Data were presented as mean ± standard error of mean (SEM). Statistical analyses were executed and graphs created with GraphPad Prism (GraphPad Software Inc., La Jolla, California, United States). *P*-values ≤ 0.05 were considered statistically significant.

## Results

### Participant Characteristics

We included 13 participants of which 11 (5 males/6 females) completed the study: age 31.0 ± 1.7 years, BMI 24.0 ± 0.6 kg/m^2^ and fat mass (%) 24.0 ± 0.6. One participant withdrew because of (unrelated) health problems whereas another participant was unable to schedule the study visits. The (self-reported) caloric intake did not differ between the three meal and one meal period at day 5 (3 meals/day 2,391 ± 211 vs. 1 meal/day 2,342 ± 233 kcal/day, *p* > 0.05), day 8 (3 meals/day 2,421 ± 208 vs. 1 meal/day 2,245 ± 305 kcal/day, *p* > 0.05) and day 11 (3 meals/day 2,404 ± 248 vs. 1 meal/day 2,307 ± 289 kcal/day, *p* > 0.05).

### Metabolic Health

The 1 meal/day pattern decreased total body mass (*p* = 0.03) and fat mass (*p* = 0.049) relative to baseline (Table 1). There were no intervention-related differences in lean mass, FFM or bone mineral density (BMD) (Table 1).

**TABLE 1:** Effect of meal frequency pattern on metabolic parameters.

DXA (*N* = 11)		
**Difference**	**3 meals/day**	**1 meal/day**
Total body weight (kg)	–0.5 ± 0.3	–1.4 ± 0.3*
Fat mass (kg)	–0.1 ± 0.2	–0.7 ± 0.2*
Lean mass (kg)	–0.3 ± 0.3	–0.7 ± 0.3
Fat-free mass (kg)	–0.3 ± 0.3	–0.7 ± 0.3
Bone mineral density (g/cm^2^)	0.003 ± 0.01	–0.005 ± 0.01
**Resting energy expenditure (*N* = 11)**	**3 meals/day**	**1 meal/day**
REE (kcal/day)	1,919 ± 146	1,778 ± 86
RQ	0.84 ± 0.02	0.82 ± 0.01
**Plasma lipids and transaminases (*N* = 11)**	**3 meals/day**	**1 meal/day**
Triglycerides (mM)	0.7 ± 0.1	0.6 ± 0.0
Cholesterol (mM)	4.2 ± 0.3	4.5 ± 0.2
HDL-C (mM)	1.6 ± 0.2	1.6 ± 0.1
LDL-C (mM)	2.4 ± 0.2	2.8 ± 0.2*
Triglycerides/HDL-C ratio	0.5 ± 0.1	0.4 ± 0.03
AST (U/L)	24.1 ± 1	29 ± 2
ALT (U/L)	28 ± 3	33 ± 5

*p ≤ 0.05 assessed with a paired t-test when data was normally distributed or otherwise the Wilcoxon sign-ranked test.

*DXA, dual-energy X-ray absorptiometry; N, number of participants included in statistical analysis and displayed in table; REE, resting energy expenditure; RQ, respiratory quotient; HDL-C, high density lipoprotein cholesterol; LDL-C, low density lipoprotein cholesterol; AST, aspartate aminotransferase; ALT, alanine aminotransferase. Data is presented as mean ± standard error of mean (SEM).*

**FIGURE 2:**
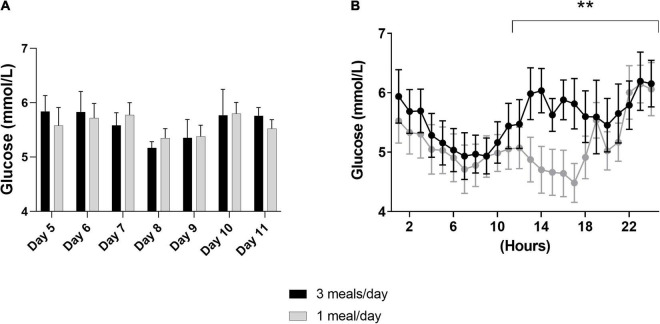
Results of the continuous glucose monitoring data from both intervention periods. In a randomized crossover design, 11 healthy participants consumed all the calories needed for a stable weight in either three meals per day (3 meals/day, breakfast, lunch and dinner) or one meal per day (1 meal/day, between 17:00 and 19:00) for 11 days. Subcutaneous glucose concentrations were measured from day 5 to day 1,111. **(A)** Displays the mean of the overall glucose concentrations per hour from day 5 until day 11, whereas, **(B)** displays the glucose concentrations measured at day 9 (*N* = 9). Data are presented as mean ± SEM. **p < 0.01 assessed with two-way repeated measures ANOVA, *N* = number of participants included in statistical analyses and displayed in graphs.

First, we investigated the effects of meal frequency on the overall mean glucose concentrations per day, which were similar in both groups (Figure 2A). Moreover, we did not find differences in the per hour glucose concentrations (day 9, Figure 2B). However, glucose concentrations were lower between 12:00 and 24:00 h in the 1 meal/day pattern (meal frequency*hours: *p*–0.009, Figure 2B).

**FIGURE 3:**
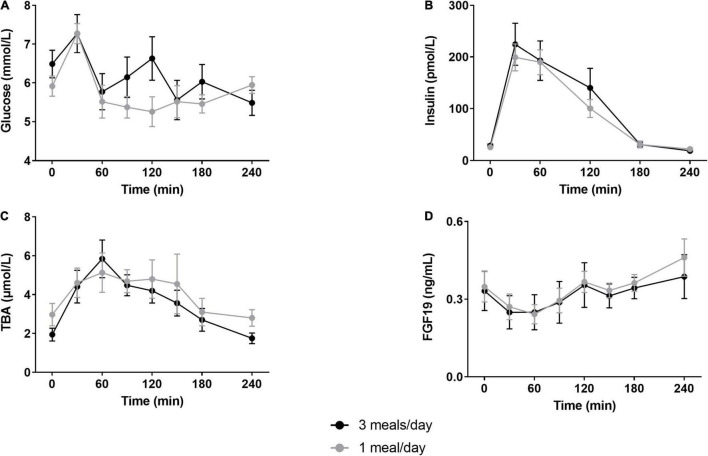
Postprandial glucose, insulin, total bile acids and fibroblast growth factor-19 responses. Participants underwent a mixed meal test after 12 days of either three meals per day (3 meals/day, breakfast, lunch and dinner) or one meal per day (1 meal/day, between 17:00 and 19:00). Postprandial responses of **(A)** glucose (*N* = 7), **(B)** insulin (*N* = 10), **(C)** total bile acids (*N* = 10) and **(D)** fibroblast growth factor 19 (*N* = 10) were unaffected by the reduced meal frequency (two-way repeated measures ANOVA, all *p* > 0.05). Data are presented as mean ± SEM. TBA, total bile acids; FGF19, fibroblast growth factor 19; *N*, number of participants included in statistical analyses and displayed in graphs.

The 1 meal/day pattern did not affect fasted REE or RQ compared to 3 meals/day measurements (Table 1). Fasted and postprandial glucose and insulin concentrations were unchanged (Figures 3A,B). We did not find changes in HOMA-IR (3 meals/day 1.3 ± 0.2 vs. 1 meal/day 1.1 ± 0.1, *p* > 0.05) or whole-body insulin sensitivity (Matsuda index 3 meals/day 13.3 ± 0.8 vs. 1 meal/day 13.9 ± 1.6, *p* > 0.05). Furthermore, fasted and postprandial bile acid and FGF19 concentrations were unaltered by the reduced meal frequency (Figures 3C,D). Fasting plasma LDL-C concentrations were increased after the 1 meal/day pattern (*p* = 0.02) whereas the other lipids, AST and ALT concentrations were unchanged (Table 1).

### Physical Performance

**FIGURE 4:**
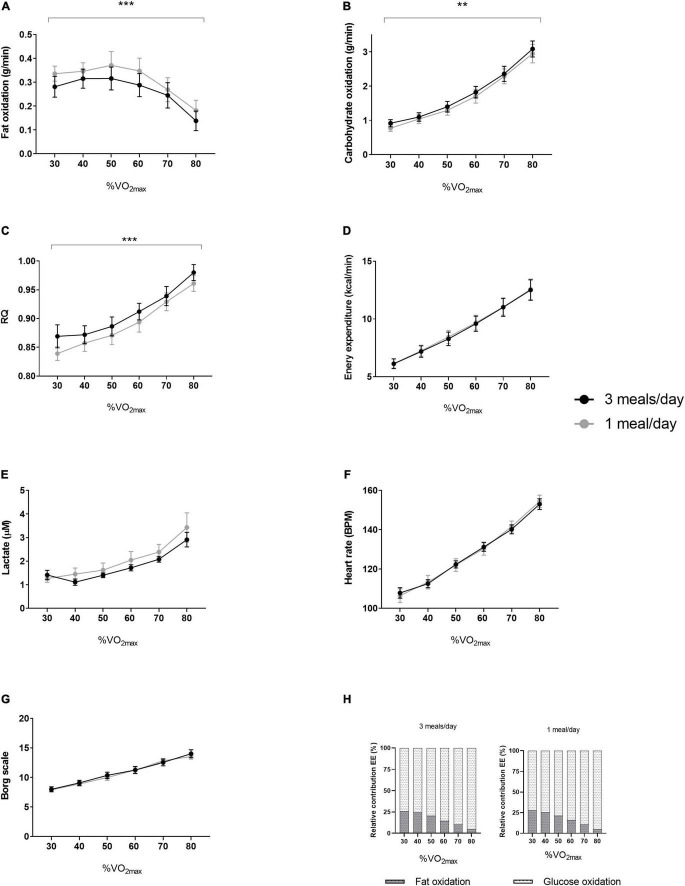
Results of the fat oxidation tests from both intervention periods. In a randomized crossover design, 11 healthy participants consumed all the calories needed for a stable weight in either three meals per day (3 meals/day, breakfast, lunch, and dinner) or one meal per day (1 meal/day, between 17:00 and 19:00) for 11 days. The fat oxidation tests were performed at day 10 of both intervention periods. Results (*N* = 11) of **(A)** fat oxidation, **(B)** carbohydrate oxidation, **(C)** RQ, **(D)** energy expenditure, **(E)** lactate, **(F)** heart rate, **(G)** Borg scale and **(H)** relative contribution of fat- and glucose oxidation to energy expenditure. Data are presented as mean ± SEM. **p < 0.01, ***p ≤ 0.001 assessed with two-way repeated measures ANOVA (*N* = 11). RQ, respiratory quotient; BPM, beats per minute; VO_2m*ax*_, maximal oxygen uptake volume. N, number of participants included in statistical analyses and displayed in graphs.

We did not detect any difference between sedentary-, low-, or MVPA intensity or total worn time (Table 2). The 1 meal/day pattern increased fat oxidation (*p* = 0.0002) and decreased glucose oxidation (*p* = 0.001) during the fat oxidation test (Figures 4A,B). In line with the increased fat oxidation, we found a decreased RQ (*p* < 0.001, Figure 4C). We did not detect differences in energy expenditure, plasma lactate concentrations, heart rate (BPM) or perceived exhaustion (Borg scale) (Figures 4D–G), nor in the relative contribution of substrate oxidation to energy expenditure (Figure 4H) between the two dietary interventions. Maximal power production did not differ between treatments (Table 2). No differences were observed during maximal oxygen uptake with respect to VO_2_ (mL/min), power (watt), lactate concentrations or perceived exhaustion (Table 2). We did not find a significant effect on jump height, isokinetic and isometric strength (Table 2).

**TABLE 2:** Effect of 1 meal-per-day pattern on physical performance.

Physical activity (*N* = 8)	3 meals/day	1 meal/day		
Sedentary (min)	480 ± 22	486 ± 34		
Low intensity (min)	223 ± 22	234 ± 24		
Total MVPA (min)	59 ± 12	62 ± 8		
Total time worn (min)	761 ± 43	782 ± 52		
**VO_2_ max (*N* = 11)**	**3 meals/day**	**1 meal/day**		
Total VO_2_ (ml/min)	3,414 ± 248	3,359 ± 225		
VO_2_ (ml/min/kg)	47 ± 3	46 ± 2		
watt maximal (watt)	286 ± 18	282 ± 16		
Perceived exhaustion (Borg scale)	19 ± 0.5	19 ± 04		
Lactate (mM)	10.3 ± 0.5	8.9 ± 1.0		
**Strength (*N* = 11)**	**3 meals/day**		**1 meal/day**	
	Pre	Post	Pre	Post
Jump height by velocity (cm)	29.5 ± 2.7	30.4 ± 2.7	29.5 ± 2.7	30.1 ± 2.7
Maximal isokinetic strength 60°/s (Nm)	190 ± 18	182 ± 18	183 ± 18	181 ± 17
Maximal isokinetic strength 120°/s (Nm)	147 ± 15	147 ± 15	148 ± 15	144 ± 14
Maximal isokinetic strength 180°/s (Nm)	120 ± 12	120 ± 12	119 ± 13	116 ± 12
Maximal isometric strength with 60° (Nm)	247 ± 17	244 ± 18	240 ± 20	237 ± 17
Wingate (max. watt)	x	842 ± 55	x	835 ± 49

*VO_2_, oxygen volume; N, number of participants included in statistical analyses and displayed in graphs; x, not assessed. Physical activity and VO_2_ were assessed with a paired T-test when data was normally distributed or otherwise the Wilcoxon sign-ranked test. Strength was assessed with the two-way repeated measurements. Data is presented as mean ± standard error of mean (SEM).*

## Discussion

This randomized crossover nutritional intervention study showed that intended eucaloric meal frequency reduction to one meal per day in the evening, lowered body- and fat mass and increased fatty acid oxidation during exercise, without impairment of aerobic capacity or strength. Furthermore, we found decreased plasma glucose concentrations during the second half of the day.

The loss of body- and fat mass after TRF (with different protocols i.e., 4- and 6-h TRF regimens and 16/8) have been reported previously (Moro et al., 2016; Cienfuegos et al., 2020). This may be the result of enhanced fat utilization throughout the day caused by lower insulin concentrations (Knapik et al., 1988; Tinsley and La Bounty, 2015). This hypothesis is supported by the observation of increased fat oxidation during exercise after the one meal per day period. Although we did not detect significant differences in caloric intake between the intervention periods, it was plausible that there was a caloric deficit of ∼110 kcal/day (1,210 kcal in 11 days) during the one meal per day intervention. Nevertheless, this caloric deficit cannot explain the total loss in body mass (–1.4 kg) and/or fat mass (–0.7 kg) at day 11, since ∼1,210 kcal account for approximately 140 gram fat. Therefore, these findings suggest that a 22 h fast in combination with the preserved daily physical activity and unchanged resting energy expenditure, caused a larger negative energy balance compared to the three meal per day pattern. This is supported by our finding of decreased plasma glucose concentrations throughout the day. However, we did not measure 24 h calorimetry, the golden standard for metabolic flexibility. It could be possible that the participants changed the macronutrient composition of their meals to maintain isocaloric intake during the one meal per day period. Moreover, we cannot exclude the beneficial role of ketone bodies nor the changes in the gut microbiome (Jamshed et al., 2019; Currenti et al., 2021a,b). Furthermore, we did not observe a decrease in lean mass during the one meal period reduction intervention, which may suggest that there was no increased net protein catabolism (e.g., decreased protein intake or use of amino acids for gluconeogenesis). However, it is possible that participants ate more during the 3 meals/day condition—which was undetected by the dietary records due to limitations of this method—to account for a notable portion of the body mass and fat mass difference.

Both bile acids and FGF19 concentrations are still increased 4–5 h after food intake (Van Nierop et al., 2019; Meessen et al., 2020). So, from an evolutionary point of view, these prolonged postprandial signals suggest that humans are well prepared for periods with limited food availability. Bile acids are released after food intake and with a function as postprandial signaling molecules within tissues of the enterohepatic cycle by activation of the intestinal and hepatic bile acid receptors such as farnesoid X receptor (FXR). FXR activation results in the production of FGF19 in the ileum, and FGF19 reaches the liver via the portal vein where it exerts unique (e.g., stimulation of fatty acid oxidation) and insulin-like actions (e.g., inhibition of gluconeogenesis, stimulation of glycogen synthesis (Kir et al., 2011; Potthoff et al., 2011). We did not observe changes in fasted- or postprandial bile acid and FGF19 concentrations. This may be explained by the fact that the duration of the overnight fast before the MMT was identical for both interventions, so we may have missed acute effects of TRF on bile acid- and FGF19 levels. Alternatively, skipping two meals may have not changed intestinal transit time or gallbladder kinetics, both important factors that determinate plasma bile acid concentrations (Sips et al., 2018; Meessen et al., 2020).

Increased cholesterol and LDL-C levels are associated with cardiovascular disease (CVD) (Levine et al., 1995). We detected a small increase in LDL-C during the one meal period, whereas the other lipid profiles were unchanged. A previous study reported that one meal per day resulted in increased total cholesterol, HDL-C and LDL-C concentrations, whereas TGs were decreased (Stote et al., 2007). They could not link their findings to dietary fat intake since they controlled food intake. In our study, participants consumed all the calories needed to maintain a stable weight within 2 h. This may have caused a higher hepatic lipid load, which in turn inhibited LDL-C receptor expression and subsequently resulted in elevated plasma LDL-C concentrations (Mustad et al., 1997). Moreover, prolonged fasting in our study could have decreased LDL catabolism in the liver to save as a nutrient reservoir to peripheral tissues (Saävendahl and Underwood, 1999). Importantly, mean plasma glucose concentrations through the day was in the one-daily meal condition, which may an health benefit. This will be particular important if the same will be the case in dysglycaemic and patients with diabetes.

We did not observe impaired physical performance as a result of the reduced meal frequency. Other studies investigating the effects of TRF on physical performance produced conflicting data. Recently, Correia et al. (2020) and Zouhal et al. (2020) reviewed the effects of exercise training and fasting and showed that TRF studies predominantly report no changes or impairment of aerobic, anaerobic and strength performance. Only a few studies showed modest improvements in VO_2m*ax*_ and strength (Tinsley and La Bounty, 2015; Bouguerra et al., 2017). Most of these studies were conducted during the Ramadan, where (unlimited) food intake between dusk and dawn is an important confounder. These kind of endurance performances are likely to be affected by the one meal per day pattern because the active muscle primarily oxidize endogenous carbohydrates (e.g., a rate-limiting step in performance), while fasting depletes these glycogen stores (Soeters et al., 2012; Hawley and Leckey, 2015).

In our study, participants were allowed to ingest their food between 17:00 and 19:00 during the one meal pattern. It is possible that our results would have been different when they were allowed to eat only in the morning, e.g., between 08:00 and 10:00. Early morning TRF increases insulin sensitivity and improves β-cell function and mid-day TRF reduces body weight and fasting glucose and insulin concentrations (Moro et al., 2016; Jones et al., 2020). Conversely, TRF in the afternoon or evening even induced glucose intolerance (Carlson et al., 2007). In the present study, the meal test did not reveal any differences in glucose, insulin, FGF19 or bile acids between the interventions. These data show that the human body can cope with one daily large meal providing all the required energy, without deteriorating metabolic regulation.

A recent review has suggested that lean mass and is generally maintained during TRF including Ramadan period (Keenan et al., 2020) supporting the data in the present study. Importantly, the participants in the present study were not competitive athletes and LBM may be easier to maintain compared to athletes. However, Moro et al. (2020) implemented TRF on elite cyclists and found a small reduction in body fat and maintained LBM. Importantly, Moro showed that power output per kg body weight increased. Brady et al. (2021) also reported reduction in body mass by TRF but did not reduced endurance performance.

The most important limitation of our study is that the study was conducted in free-living healthy individuals. Data on food intake was obtained with food diaries completed by the participants. Nonetheless, our results showed that the participants were most likely compliant to the one meal per day pattern since the glucose sensor showed lower glucose concentrations throughout the day. It is a strong point that we carefully characterized strength, maximal power, and aerobic capacity under controlled conditions and with state-of-the art methodologies.

In summary, we found that one meal per day in the evening lowers body weight and adapts metabolic flexibility during exercise via increased fat oxidation, whereas the physical performance and the postprandial responses to liquid mixed meals were unaffected. Future studies should investigate the effect of TRF in participants with reduced metabolic flexibility (e.g., patients with obesity, metabolic syndrome or type 2 diabetes mellitus). Moreover, the timing of the one meal consumption in early morning may yield larger effects.

## Data Availability Statement

The raw data supporting the conclusions of this article will be made available by the authors, without undue reservation.

## Ethics Statement

The studies involving human participants were reviewed and approved by the Ethics Committee at Norwegian School of Sport Sciences (Application #56-190618). The patients/participants provided their written informed consent to participate in this study.

## Author Contributions

EM co-designed the study, executed statistical analyses, and wrote the manuscript. HA co-designed the study, performed clinical experiments, executed statistical analyses, and reviewed the manuscript. TB and AR performed clinical experiments, executed statistical analyses, and reviewed the manuscript. EJ performed clinical experiments and reviewed the manuscript. AK was responsible MD during study and reviewed the manuscript. SO and FS performed laboratory analyses and reviewed the manuscript. EK, JR and AK reviewed the manuscript. JJ and MS designed the study, reviewed, and edited the manuscript. All authors contributed to the article and approved the submitted version.

## Conflict of Interest

The authors declare that the research was conducted in the absence of any commercial or financial relationships that could be construed as a potential conflict of interest.

## Publisher’s Note

All claims expressed in this article are solely those of the authors and do not necessarily represent those of their affiliated organizations, or those of the publisher, the editors and the reviewers. Any product that may be evaluated in this article, or claim that may be made by its manufacturer, is not guaranteed or endorsed by the publisher.

## Abbreviations

ALT: Alanine amino transferase
AST: Aspartate amino transferase
BMC: Bone mineral content
BMD: Bone mineral density
BMI: Body mass index
CGM: Continuous glucose monitoring system
CPM: Counts per minute
CV: Coefficient of Variance
DXA: Dual-energy X-ray absorptiometry
FFM: Fat free mass
FGF19: Fibroblast growth factor 19
FXR: Farnesoid X receptor
HDL-C: High-density lipoprotein cholesterol
LDL-C: Low-density lipoprotein cholesterol
HOMA-IR: Homeostatic model assessment for insulin resistance
MMT: Mixed meal test
MVPA: Moderate-to-vigorous physical activity
REE: Resting energy expenditure
RPM: Revolutions per minute
RQ: Respiratory quotient
TGR5: Takeda G protein coupled 5 receptor
TRF: Time-restricted feeding
TGs: Triglycerides
T: Time point
T2DM: Type 2 diabetes mellitus
VCO_2_: Carbon dioxide volume
VO_2_: Oxygen volume
VO_2m*ax*_: Maximal oxygen uptake.

## References

[B1] Andersson Hall U. Edin F. Pedersen A. Madsen K. (2016). Whole-body fat oxidation increases more by prior exercise than overnight fasting in elite endurance athletes. *Appl. Physiol. Nutr. Metab.* 41 430–437. 10.1139/apnm-2015-0452 26988766

[B2] Beregning av kaloribehov—NHI.no (2021). Available online at: https://nhi.no/skjema-og-kalkulatorer/kalkulatorer/diverse/beregning-av-kaloribehov/ (accessed June 11, 2020).

[B3] Borg G. A. (1982). Psychophysical bases of perceived exertion. *Med. Sci. Sports Exerc.* 14 377–381. 7154893

[B4] Bouguerra L. Ben Abderrahman A. Chtourou H. Zouhal H. Tabka Z. Prioux J. (2017). The effect of time-of-day of training during Ramadan on physiological parameters in highly trained endurance athletes. *Biol. Rhythm Res.* 48 541–555. 10.1080/09291016.2016.1276271

[B5] Brady A. J. Langton H. M. Mulligan M. Egan B. (2021). Effects of 8 wk of 16:8 time-restricted eating in male middle- and long-distance runners. *Med. Sci. Sports Exerc.* 53 633–642. 10.1249/MSS.0000000000002488 32796255

[B6] Carlson O. Martin B. Stote K. S. Golden E. Maudsley S. Najjar S. S. (2007). Impact of reduced meal frequency without caloric restriction on glucose regulation in healthy, normal-weight middle-aged men and women. *Metabolism* 56 1729–1734. 10.1016/J.METABOL.2007.07.018 17998028 PMC2121099

[B7] Cienfuegos S. Gabel K. Kalam F. Ezpeleta M. Wiseman E. Pavlou V. (2020). Effects of 4- and 6-h time-restricted feeding on weight and cardiometabolic health: a randomized controlled trial in adults with obesity. *Cell Metab.* 32 366.e3–378.e3. 10.1016/J.CMET.2020.06.018 32673591 PMC9407646

[B8] Correia J. M. Santos I. Pezarat-Correia P. Minderico C. Schoenfeld B. J. Mendonca G. V. (2021). Effects of time-restricted feeding on supramaximal exercise performance and body composition: a randomized and counterbalanced crossover study in healthy men. *Int. J. Environ. Res. Public Health* 18:7227. 10.3390/ijerph18147227 34299702 PMC8303210

[B9] Correia M. J. Santos I. Pezarat-Correia P. Minderico C. Mendonca G. V. (2020). Effects of Intermittent fasting on specific exercise performance outcomes: a systematic review including meta-analysis. *Nutrients* 12:1390. 10.3390/nu12051390 32408718 PMC7284994

[B10] Currenti W. Buscemi S. Cincione R. I. Cernigliaro A. Godos J. Grosso G. (2021a). Time-Restricted feeding and metabolic outcomes in a cohort of italian adults. *Nutrients* 13:1651 10.3390/nu13051651 34068302 PMC8153259

[B11] Currenti W. Godos J. Castellano S. Caruso G. Ferri R. Caraci F. (2021b). Association between time restricted feeding and cognitive status in older italian adults. *Nutrients* 13:191. 10.3390/nu13010191 33435416 PMC7827225

[B12] Dahl M. A. Areta J. L. Jeppesen P. B. Birk J. B. Johansen E. I. Ingemann-Hansen T. (2020). Co-ingestion of protein and carbohydrate in the early recovery phase improves endurance performance despite like glycogen degradation and AMPK phosphorylation. *J. Appl. Physiol.* 129 297–310. 10.1152/japplphysiol.00817.2019 32584664

[B13] Duarte J. P. Valente-Dos-Santos J. Coelho-E-Silva M. J. Couto P. Costa D. Martinho D. (2018). Reproducibility of isokinetic strength assessment of knee muscle actions in adult athletes: torques and antagonist-agonist ratios derived at the same angle position. *PLoS One* 13:e0202261. 10.1371/journal.pone.0202261 30110385 PMC6093703

[B14] Frayn K. (1983). Calculation of substrate oxidation from gaseous exchange rates in vivo. *J. Appl. Physiol.* 55 628–634. 10.1152/jappl.1983.55.2.628 6618956

[B15] Habets B. Staal J. B. Tijssen M. van Cingel R. (2018). Intrarater reliability of the Humac NORM isokinetic dynamometer for strength measurements of the knee and shoulder muscles. *BMC Res. Notes* 11:15. 10.1186/s13104-018-3128-9 29321059 PMC5764011

[B16] Halberg N. Henriksen M. Söderhamn N. Stallknecht B. Ploug T. Schjerling P. (2005). Effect of intermittent fasting and refeeding on insulin action in healthy men. *J. Appl. Physiol.* 99 2128–2136. 10.1152/japplphysiol.00683.2005 16051710

[B17] Hansen B. H. Kolle E. Dyrstad S. M. Holme I. Anderssen S. A. (2012). Accelerometer-determined physical activity in adults and older people. *Med. Sci. Sports Exerc.* 44 266–272. 10.1249/MSS.0b013e31822cb354 21796052

[B18] Hawley J. A. Leckey J. J. (2015). Carbohydrate dependence during prolonged, intense endurance exercise. *Sports Med.* 45(Suppl. 1) S5–S12. 10.1007/s40279-015-0400-1 26553495 PMC4672006

[B19] Jamshed H. Beyl R. A. Della Manna D. L. Yang E. S. Ravussin E. Peterson C. M. (2019). Early time-restricted feeding improves 24-hour glucose levels and affects markers of the circadian clock, aging, and autophagy in humans. *Nutrients* 11:1234. 10.3390/nu11061234 31151228 PMC6627766

[B20] Jones R. Pabla P. Mallinson J. Nixon A. Taylor T. Bennett A. (2020). Two weeks of early time-restricted feeding (eTRF) improves skeletal muscle insulin and anabolic sensitivity in healthy men. *Am. J. Clin. Nutr.* 112 1015–1028. 10.1093/ajcn/nqaa192 32729615 PMC7528549

[B21] Kant A. K. (2018). Eating patterns of US adults: meals, snacks, and time of eating. *Physiol. Behav.* 193 270–278. 10.1016/j.physbeh.2018.03.022 29574043

[B22] Keenan S. Cooke M. B. Belski R. (2020). The effects of intermittent fasting combined with resistance training on lean body mass: a systematic review of human studies. *Nutrients* 12:2349. 10.3390/nu12082349 32781538 PMC7468742

[B23] Kir S. Beddow S. A. Samuel V. T. Miller P. Previs S. F. Suino-Powell K. (2011). FGF19 as a postprandial, insulin-independent activator of hepatic protein and glycogen synthesis. *Science* 331 1621–1624. 10.1126/science.1198363 21436455 PMC3076083

[B24] Knapik J. J. Meredith C. N. Jones B. H. Suek L. Young V. R. Evans W. J. (1988). Influence of fasting on carbohydrate and fat metabolism during rest and exercise in men. *J. Appl. Physiol.* 64 1923–1929. Available online at: https://journals.physiology.org/doi/abs/10.1152/jappl.1988.64.5.1923 (accessed May 22, 2020). 10.1152/jappl.1988.64.5.1923 3292504

[B25] Kopp W. (2019). How western diet and lifestyle drive the pandemic of obesity and civilization diseases. *Diabetes Metab. Syndr. Obes.* 12 2221–2236. 10.2147/DMSO.S216791 31695465 PMC6817492

[B26] Lafontan M. Langin D. (2009). Lipolysis and lipid mobilization in human adipose tissue. *Prog. Lipid Res.* 48 275–297. 10.1016/J.PLIPRES.2009.05.001 19464318

[B27] Levine G. N. Keaney J. F. Vita J. A. (1995). Cholesterol reduction in cardiovascular disease-clinical benefits and possible mechanisms. *N. Engl. J. Med.* 332 512–521. 10.1056/NEJM199502233320807 7830734

[B28] Manson J. E. Skerrett P. J. Greenland P. VanItallie T. B. (2004). The escalating pandemics of obesity and sedentary lifestyle. *Arch. Intern. Med.* 164:249. 10.1001/archinte.164.3.249 14769621

[B29] Matsuda M. DeFronzo R. A. (1999). Insulin sensitivity indices obtained from oral glucose tolerance testing: comparison with the euglycemic insulin clamp. *Diabetes Care* 22 1462–1470. 10.2337/diacare.22.9.1462 10480510

[B30] McAllister M. J. Pigg B. L. Renteria L. I. Waldman H. S. (2020). Time-restricted feeding improves markers of cardiometabolic health in physically active college-age men: a 4-week randomized pre-post pilot study. *Nutr. Res.* 75 32–43. 10.1016/j.nutres.2019.12.001 31955013

[B31] Meessen E. C. E. Sips F. L. P. Eggink H. M. Koehorst M. Romijn J. A. Groen A. K. (2020). Model-based data analysis of individual human postprandial plasma bile acid responses indicates a major role for the gallbladder and intestine. *Physiol. Rep.* 8:e14358. 10.14814/phy2.14358 32170845 PMC7070101

[B32] Meiselman H. L. (2000). *Dimensions Of The Meal: The Science, Culture, Business, And Art Of Eating.* Washington, D.C: Aspen Publishers, Inc. Available online at: https://agris.fao.org/agris-search/search.do?recordID=US201300044234 (accessed October 17, 2021).

[B33] Moon S. Kang J. Kim S. H. Chung H. S. Kim Y. J. Yu J. M. (2020). Beneficial effects of time-restricted eating on metabolic diseases: a systemic review and meta-analysis. *Nutrients* 12:1267. 10.3390/nu12051267 32365676 PMC7284632

[B34] Moro T. Tinsley G. Bianco A. Marcolin G. Pacelli Q. F. Battaglia G. (2016). Effects of eight weeks of time-restricted feeding (16/8) on basal metabolism, maximal strength, body composition, inflammation, and cardiovascular risk factors in resistance-trained males. *J. Transl. Med.* 14:290. 10.1186/s12967-016-1044-0 27737674 PMC5064803

[B35] Moro T. Tinsley G. Longo G. Grigoletto D. Bianco A. Ferraris C. (2020). Time-restricted eating effects on performance, immune function, and body composition in elite cyclists: a randomized controlled trial. *J. Int. Soc. Sports Nutr.* 17:65. 10.1186/s12970-020-00396-z 33308259 PMC7733258

[B36] Mustad V. A. Etherton T. D. Cooper A. D. Mastro A. M. Pearson T. A. Jonnalagadda S. S. (1997). Reducing saturated fat intake is associated with increased levels of LDL receptors on mononuclear cells in healthy men and women. *J. Lipid Res* 38 459–468. 10.1016/s0022-2275(20)37254-0 9101427

[B37] Paoli A. Tinsley G. Bianco A. Moro T. (2019). The influence of meal frequency and timing on health in humans: the role of fasting. *Nutrients* 11:719. 10.3390/nu11040719 30925707 PMC6520689

[B38] Patterson R. E. Sears D. D. (2017). Metabolic effects of intermittent fasting. *Annu. Rev. Nutr.* 37 371–393. 10.1146/annurev-nutr-071816-064634 28715993 PMC13170603

[B39] Pellegrini M. Cioffi I. Evangelista A. Ponzo V. Goitre I. Ciccone G. (2020). Effects of time-restricted feeding on body weight and metabolism. A systematic review and meta-analysis. *Rev. Endocr. Metab. Disord.* 21 17–33. 10.1007/s11154-019-09524-w 31808043

[B40] Potthoff M. J. Boney-Montoya J. Choi M. He T. Sunny N. E. Satapati S. (2011). FGF15/19 regulates hepatic glucose metabolism by inhibiting the CREB-PGC-1α pathway. *Cell Metab.* 13 729–738. 10.1016/j.cmet.2011.03.019 21641554 PMC3131185

[B41] Rustad P. I. Sailer M. Cumming K. T. Jeppesen P. B. Kolnes K. J. Sollie O. (2016). Intake of protein plus carbohydrate during the first two hours after exhaustive cycling improves performance the following day. *PLoS One* 11:e0153229. 10.1371/journal.pone.0153229 27078151 PMC4831776

[B42] Saävendahl L. Underwood L. E. (1999). Fasting increases serum total cholesterol, ldl cholesterol and apolipoprotein b in healthy, nonobese humans. *J. Nutr.* 129 2005–2008. 10.1093/jn/129.11.2005 10539776

[B43] Schaap F. G. van der Gaag N. A. Gouma D. J. Jansen P. L. M. (2009). High expression of the bile salt-homeostatic hormone fibroblast growth factor 19 in the liver of patients with extrahepatic cholestasis. *Hepatology* 49 1228–1235. 10.1002/hep.22771 19185005

[B44] Schrezenmeir J. Keppler I. Fenselau S. Weber P. Biesalski H. K. Probst R. (1993). The phenomenon of a high triglyceride response to an oral lipid load in healthy subjects and its link to the metabolic syndrome. *Ann. N. Y. Acad. Sci.* 683 302–314. 10.1111/j.1749-6632.1993.tb35721.x 8352452

[B45] Shetty P. S. Henry C. J. K. Black A. E. Prentice A. M. (1988). *European Journal Of Clinical Nutrition, J. Libbey.* Available online at: https://pascal-francis.inist.fr/vibad/index.php?action=getRecordDetail&idt=3029488 (accessed November 15, 2021).

[B46] Sips F. L. P. Eggink H. M. Hilbers P. A. J. Soeters M. R. Groen A. K. van Riel N. A. W. (2018). In silico analysis identifies intestinal transit as a key determinant of systemic bile acid metabolism. *Front. Physiol.* 9:631. 10.3389/fphys.2018.00631 29951001 PMC6008656

[B47] Smith R. L. Soeters M. R. Wüst R. C. I. Houtkooper R. H. (2018). Metabolic flexibility as an adaptation to energy resources and requirements in health and disease. *Endocr. Rev.* 39 489–517. 10.1210/er.2017-00211 29697773 PMC6093334

[B48] Soeters M. R. Lammers N. M. Dubbelhuis P. F. Ackermans M. Jonkers-Schuitema C. F. Fliers E. (2009). Intermittent fasting does not affect whole-body glucose, lipid, or protein metabolism. *Am. J. Clin. Nutr.* 90 1244–1251. 10.3945/ajcn.2008.27327 19776143

[B49] Soeters M. R. Soeters P. B. Schooneman M. G. Houten S. M. Romijn J. A. (2012). Adaptive reciprocity of lipid and glucose metabolism in human short-term starvation. *Am. J. Physiol. Endocrinol. Metab.* 303 E1397–E1407. 10.1152/ajpendo.00397.2012 23074240

[B50] Sollie O. Jeppesen P. B. Tangen D. S. Jernerén F. Nellemann B. Valsdottir D. (2018). Protein intake in the early recovery period after exhaustive exercise improves performance the following day. *J. Appl. Physiol.* 125, 1731–1742. 10.1152/japplphysiol.01132.2017 30212306

[B51] Stote K. S. Baer D. J. Spears K. Paul D. R. Harris G. K. Rumpler W. V. (2007). A controlled trial of reduced meal frequency without caloric restriction in healthy, normal-weight, middle-aged adults. *Am. J. Clin. Nutr.* 85 981–988. 10.1093/ajcn/85.4.981 17413096 PMC2645638

[B52] Tangen D. S. Nielsen S. R. Kolnes K. J. Jensen J. (2020). Caffeine increases vertical jumping height in young trained males before but not after a maximal effort strength training session. *J. Sci. Sport Exerc.* 2 145–153. 10.1007/s42978-020-00060-7

[B53] Thiebaud D. Jacot E. DeFronzo R. A. Maeder E. Jequier E. Felber J. P. (1982). The effect of graded doses of insulin on total glucose uptake, glucose oxidation, and glucose storage in man. *Diabetes* 31 957–963. 10.2337/diacare.31.11.957 6757014

[B54] Tinsley G. M. La Bounty P. M. (2015). Effects of intermittent fasting on body composition and clinical health markers in humans. *Nutr. Rev.* 73 661–674. 10.1093/nutrit/nuv041 26374764

[B55] Van Nierop F. S. Meessen E. C. E. Nelissen K. G. M. Achterbergh R. Lammers L. A. Vaz F. M. (2019). Differential effects of a 40 hour fast and bile acid supplementation on human GLP-1 and FGF19 responses. *Am. J. Physiol. Endocrinol. Metab.* 317 E494–E502. 10.1152/ajpendo.00534.2018 31237451

[B56] Weir J. B. de V. (1949). New methods for calculating metabolic rate with special reference to protein metabolism. *J. Physiol.* 109 1–9. 10.1113/jphysiol.1949.sp004363 15394301 PMC1392602

[B57] Zouhal H. Saeidi A. Salhi A. Li H. Essop M. F. Laher I. (2020). Exercise training and fasting: current insights. *Open Access J. Sport. Med.* 11 1–28. 10.2147/OAJSM.S224919 32021500 PMC6983467

